# The role of cross sectional imaging in the management of acute pyogenic inguinal abscess - extrapelvic versus intrapelvic origin

**DOI:** 10.1186/1471-2334-13-155

**Published:** 2013-03-27

**Authors:** Wei-Hsiu Hsu, Ching-Yu Lee, Li-Ju Lai, Tsung-Yu Huang, Kuo-Ti Peng

**Affiliations:** 1 Division of Sports Medicine, Department of Orthopedic Surgery, Chang Gung Memorial Hospital at Chia Yi, 6 West Section Chia Pu Road, Chia Yi Hsien 613, Taiwan; 2 Department of Medicine, Chang Gung University, 259 Wen-Hwa 1st Road, Kwei-Shan, Tao-Yuan 333, Taiwan

**Keywords:** Inguinal abscess, Intrapelvic origin, Extrapelvic origin, Prognostic factors

## Abstract

**Background:**

Abscesses involving the inguinal region as manifestations of complex soft-tissue infections are rare, and the infectious route is usually unclear. The purpose of this study was to ascertain the importance of imaging study and whether the clinical presentations differ between the extrapelvic and intrapelvic origin.

**Methods:**

Patients who presented with inguinal abscess between January 2003 and December 2010 were evaluated retrospectively. All patients received broad-spectrum antibiotic therapy and debridement. Imaging studies, including computed tomography or magnetic resonance imaging, were performed in all patients to elucidate the origin and extent of infectious disease, and the results were reviewed. Clinical data, laboratory examination findings, and culture results were analyzed.

**Results:**

Twenty-eight patients were enrolled in the study: 13 patients whose infections were of extrapelvic origin (Group 1) and 15 patients of intrapelvic origin (Group 2). Imaging studies yielded information that helped guiding the treatment. Gram-positive coccus infection was more frequent in Group 1 (*p* < 0.001), while mixed pathogen and anaerobic bacterial infection were more frequent in Group 2 (*p* = 0.002 and *p* = 0.006, respectively). Group 2 had a higher incidence of history of malignancy and chronic renal failure (*p* = 0.044 and *p* = 0.038, respectively).

**Conclusions:**

Computed tomography and magnetic resonance imaging are helpful in diagnosing cases of inguinal abscess and determining the extent of infection. In patients presenting with acute pyogenic inguinal abscess, a higher prevalence of chronic renal failure and history of malignancy were found in those with an intrapelvic, as compared with an extrapelvic, origin of infection.

## Background

Inguinal abscesses as manifestations of deep soft-tissue infections are complex and rare, and the infectious route is usually unclear. The inguinal region communicates with the peritoneal or retroperitoneal space and thigh through several routes, including the psoas sheath, femoral canal, sacrosciatic notch, pudendal canal, and obturator foramen [1,2]. It has been reported that inguinal abscesses may arise from peritoneal or retroperitoneal abscesses, such as ruptured appendicitis, colonic diverticulitis, and pyelonephritis [3-5]. Hence, the infection may be of extrapelvic or intrapelvic origin [6-11]. Understanding the etiology of the inguinal abscess is helpful in guiding definitive treatment and the prescription of empiric antibiotics. Inguinal abscesses resulting from extrapelvic pyomyositis usually appear as well-defined cavities, while those resulting from intrapelvic infection may be complicated by underlying bowel disease, infective spondylodiskitis, or urinary tract infection [9-20]. Empiric antibiotics and surgical planning should be guided by the pathophysiology of the disease. Computed tomography (CT) and magnetic resonance imaging (MRI) are valuable diagnostic tools. It is currently unclear whether the presentation and prognosis for patients with acute pyogenic inguinal abscess differ according to whether the infection is of extrapelvic versus intrapelvic origin.

A retrospective study was performed to investigate the clinical manifestations and outcomes in patients who presented with acute pyogenic inguinal abscess. Such information may facilitate more accurate prediction of the outcome for patients as well as guide better management of this disease. Approval for this study was obtained from the institutional review board of Chang Gung Memorial Foundation.

## Methods

### Patients

Under the approval of institutional board review of Chang Gung Memorial Foundation (100-0667B), the medial records of three hundred and forty six patients diagnosed with the ICD code 6822 (cellulitis and abscess, trunk) from Chang Gung Memorial Hospital at Chia Yi were comprehensively reviewed between January 2003 and December 2010. The inclusion criteria were a presentation groin mass associated with fever greater than 38.3 degree at emergency department that demanded surgical treatment. Twenty-eight patients diagnosed with inguinal abscess and treated with surgical debridement were included in the current study. Medical records, laboratory examination results, and imaging findings were retrospectively reviewed and analyzed. Patients with inguinal abscess presented with a swollen, erythematous, and tender inguinal mass. Most patients experienced a gradual onset of a limping gait, fever, and chills. Inguinal abscess was confirmed by CT or MRI, along with surgical findings. The CT or MRI features in patients with inguinal abscess included the following: (1) asymmetrical enlargement of the underlying affected muscle, (2) “ring sign,” or rim enhancement of the abscess wall with lower central attenuation, and (3) air bubbles.

### Methods

Upon diagnosis of an inguinal abscess, the treatment strategy included broad-spectrum antibiotic therapy, aggressive resuscitation, and adequate debridement. Microbial infection was confirmed by culture results from soft tissue or blood collected in the emergency department (ED) and during surgery. The antibiotic regimen was modified appropriately after the microbial culture results were obtained. Intensive care and aggressive resuscitation, including fluid challenge and inotropic drugs, were administered to maintain mean arterial pressure above 65 mm Hg. Surgical planning was based on imaging findings. If an extrapelvic origin of infection was identified, debridement of the inguinal abscess along with the extrapelvic site was performed by an orthopedic surgeon. If the imaging findings showed an intrapelvic origin of infection, combination surgery involving colorectal or urologic surgeons was performed.

The patients were divided into two groups for further analysis: those with an extrapelvic origin of infection (Group 1) and those with an intrapelvic origin of infection (Group 2). Extrapelvic origin was defined as that thigh represented the primary infectious focus. On the other hand, when the infectious focus was located within the pelvic cavity including intraperitoneal, retroperitoneal and genitourinary pathology, it was defined as intrapelvic origin.

Clinical parameters including age, gender, comorbidities, clinical manifestations, laboratory findings at the time of admission, bacteriologic findings, APACHE II score, length of hospital stay, and mortality were recorded and compared.

### Statistical analyses

All statistical analyses were performed using SPSS, version 10.0 (SPSS Inc, Chicago, IL). The Wilcoxon rank sum test was used for discontinuous variables, and the Fisher exact test was used for continuous variables. Statistical significance was set at a *p* value of <0.05.

## Results

### Clinical and demographic data of patients with inguinal abscess

This series consisted of 28 patients. The median patient age was 60.5 years. Nineteen patients (67.9%) were male. Most patients were immunocompromised (78.6%). Diabetes mellitus (77.3%) was the leading disease in immunocompromised patients. Computed tomography and MRI constituted good diagnostic tools in all patients.

### Group comparison of clinical characteristics

No significant differences were found between the two groups in age and gender. Although there were no differences in duration of symptoms prior to arrival at the ED, surgery was performed earlier in Group 2 patients that the time from onset of symptoms to first surgical intervention was shorter for patients in Group 2 (*p* = 0.048). Group 2 patients also presented a more toxic appearance that the mean APACHE II score 24 hours after admission were higher ( *p* = 0.022). Furthermore, the frequency of surgical debridement were higher for Group 2 (*p* = 0.009) (Table 1). However, there were no differences between Group 1 and Group 2 in total length of hospitalization or length of ICU stay, and mortality. Diabetes mellitus was both prevalent in Group 1 and Group 2, while patients with a history of malignancy and chronic renal failure were more strongly represented in Group 2 (*p* = 0.044 and *p* = 0.038, respectively).

**Table 1:** Group comparison of clinical characteristics

	**Extra-pevic origin**	**Intrapevic origin**	**p-value**
	**n=13**	**n=15**	
**Age**	57.8 (23, 89)	62.9 (52, 75)	0.613
**Gender**			
Male	9 (69.2)	9 (60)	0.705
Female	4 (30.8)	6 (40)	
**TiOA(day)**	4.4 (2, 28)	6.2 (2, 30)	0.289
**TiOS(h)**	27.3 (5, 144)	9.4 (3, 25)	0.048*
**Comorbidity**			
Diabetes mellitus	6 (41.2)	10 (66.7)	1.000
Chronic renal insufficiency	1 (7.7)	7 (46.7)	0.038*
Chronic viral hepatitis	3 (23.1)	2 (13.3)	0.634
Malignancy	0	6 (40)	0.044*
**ICU stay(patient number)**	4	10	
**APACHE II score**	16.6 (11, 26)	20.6 (13, 27)	0.022*
**ICU stay(day)**	5.8 (0,35)	6.3 (0,30)	0.499
**Hospital stay(day)**	38.2 (5, 126)	42.4 (9,86)	0.18
**Surgical procedure**			
Debridement	1.8	3.2	0.009*
Colostomy	0	6	0.044*
CT-guided drainage	0	1	
**Soft tissue reconstruction**	6	2	0.096
Rotational flap	3	0	0.087
STSG or FTSG	2	1	0.583
VAC	1	1	1.000
**Mortality**	1 (7.7)	2 (13.3)	1.000

Data are presented as median (min, max) or frequency (%).

TiOA, duration of symptoms prior to arrival to the ER.

TiOS, time of the first surgical intervention.

STSG: split thickness skin graft, FTSG, full thickness skin graft, VAC: vaccum assisted closure.

APACHE, acute physiological, age, and chronic health evaluation.

*: p < 0.05.

### Group comparison of laboratory data and microbiology

Most patients in both groups presented with leukocytosis with left shift, along with a increased C reactive protein level. Severe hypoalbuminemia was frequently observed in both groups. However, anemia and hyponatremia were more frequently observed in Group 2 (*p* = 0.024 and *p* = 0.023, respectively), which reflected the chronic deliberated status in these patients (Table 2).

**Table 2:** Group comparison of laboratory data

	**Extrapevic origin**	**Intrapevic origin**	**p-value**
	**n=13**	**n=15**	
Total WBC			
Leukocytosis (>=12000/L,)	10 (76.9)	13 (86.7)	0.640
Leutropenia (<=4000/L,)	1	1	
Leukocytosis or leutropenia	11 (84.6)	14 (93.3)	0.583
Differential count			
Band formation	7 (53.8)	12 (80)	0.227
Band >=10	0 (0)	3 (20)	0.226
Neutrophilia (>7500/L,)	12 (92.3)	14 (93.3)	1.000
Lymphocytopenia (<1000/L,)	2 (13.4)	6 (40)	0.221
Thrombocytopenia (<150000/L,)	2 (13.4)	2 (13.3)	
Hemoglobin (g/dL)	12.2 (8.0, 15.3)	10.2 (6.7, 14.2)	0.024*
C-reactive protein (mg/dL)	205.6 (15.7, 458)	262.9 (120, 412)	0.111
Glucose (mg/dL)	153.8 (91, 249)	220.3 (81, 497)	0.121
Sodium (meq/L)	137.6 (124, 149)	131.7 (122, 137)	0.023*
ALT (u/L)	48.9 (15, 85)	34.6 (14, 187)	0.028*
Hypoalbuminemia (<2 g/dL)	5(38.4)	10 (66.7)	0.255
Bacteremia	3 (23.1)	2 (13.3)	0.640
Bacteriological findings			
Positive blood culture	1	1	1.000
Positive wound culture	11	13	1.000
Positive blood and wound culture	2	1	0.096
GPC	10 (76.9)	1 (6.7)	<0.001*
GNB	2 (15.4)	2 (13.3)	1.000
Mixed infection	1 (7.7)	9 (60)	0.006*
Anaerobes	0	8 (53.3)	0.002*

Data are presented as median (min, max) or frequency (%).

GPC, gram positive cocci, GNB, gram negative bacilli.

*:p < 0.05.

Gram-positive coccus infection was more frequent in Group 1 (*p* < 0.001), while mixed pathogen and anaerobic bacterial infection were more common in Group 2 (*p* = 0.002 and *p* = 0.006, respectively). In patients with inguinal abscess of intrapelvic origin, *Escherichia coli* was the most common pathogen (40%), followed by *Bacteroides fragilis* (33.3%). *Staphylococcus aureus* was the most common pathogen (69.2%) in those with infection of extrapelvic origin, and the occurrence of oxacillin-resistant staphylococcal infection was predominant (33.3%) (Table 3).

**Table 3:** Summary of microbiology

**Microbial pathogens**	**Extraplevic origin (n=13)**	**Intrapelvic origin (n=15)**
**Gram positive pathogen**		
*Staphylococcus aureus:*		
-MSSA	5	3
-MRSA	4	0
*Streptococcus:*		
Alpha-hemolytic-		
*-S. viridans*	1	2
Beta-hemolytic-		
*-S. pyogenes*	2	0
-Group B *Streptococcus*		
*Enterococcus faecalis*		2
**Gram negative pathogen**		
*Kleb.pneumoniae*	2	2
*Pseudomonas Aeruginosa*	0	2
E. Coli	0	6
Anaerobes		
*Bacteroides fragilis*	0	5
*Clostridium perfringens*	0	1
*Proteus mirabilis*	0	2
*Prevotella* spp.	0	2
**Mixed infection**	1	9

MSSA, Methicillin-susceptible S. aureus.

MRSA, Methicillin-resistant S. aureus.

One patient in each group died in severe sepsis and multi-organ failure despite of broad spectrum antibiotics and surgical debridment.

## Discussion

**Figure 1:**
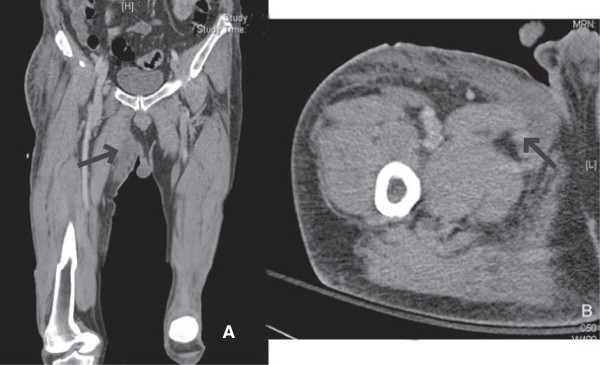
**Computed tomography of the pelvic area and right thigh showed formation of an abscess with gas bubbles at the medial portion of the right thigh that involved the underlying adductor muscle.** (**A**), Coronal section, (**B**), Axial section.

**Figure 2:**
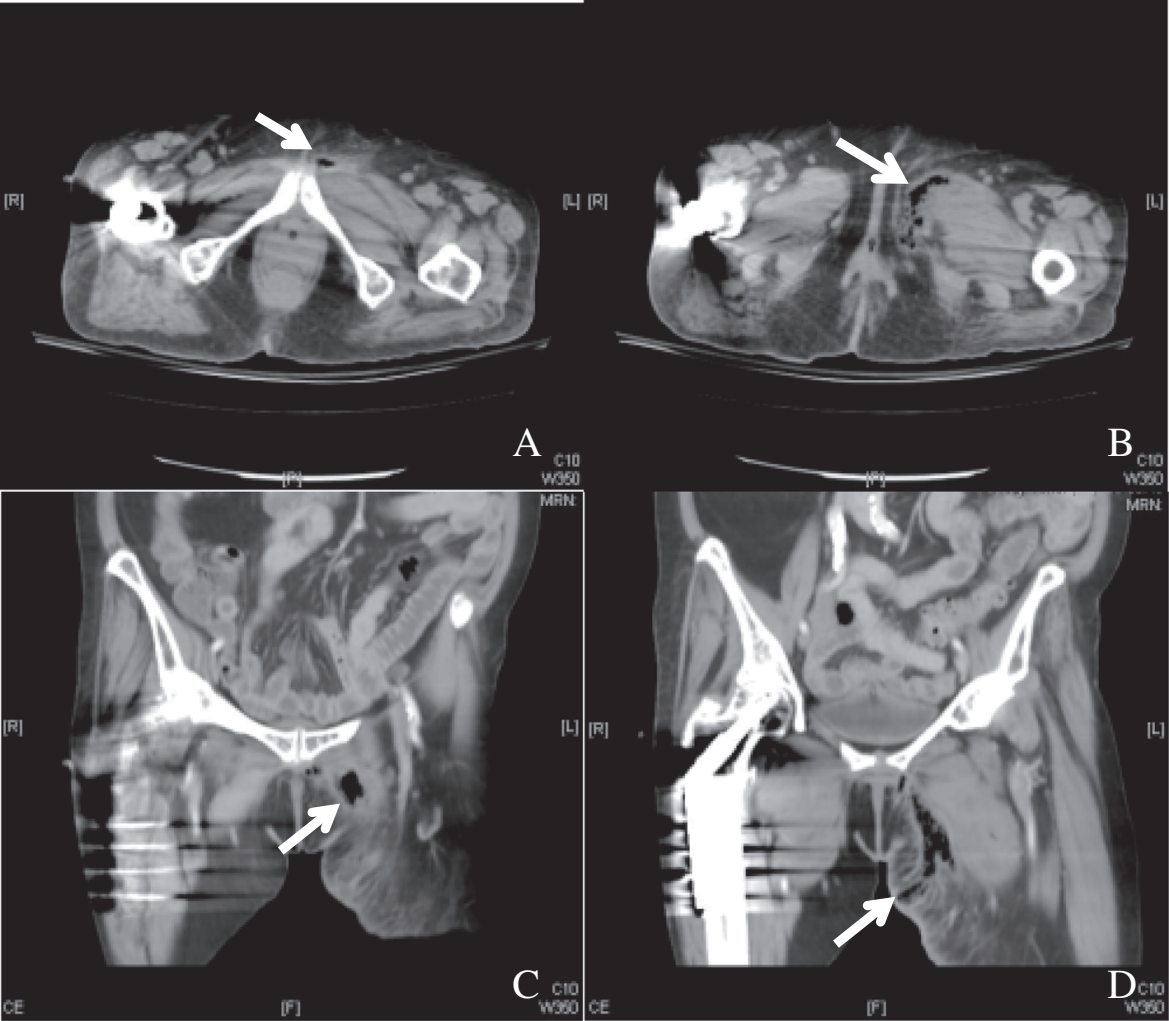
**Computed tomography of the pelvic area and left thigh showed formation of an abscess with gas bubbles at groin area.** Extension to the medial portion of the left thigh with blurring of the underlying adductor muscle was demonstrated. (**A**,**B**), Axial section, (**C**.**D**), Coronal section.

**Figure 3:**
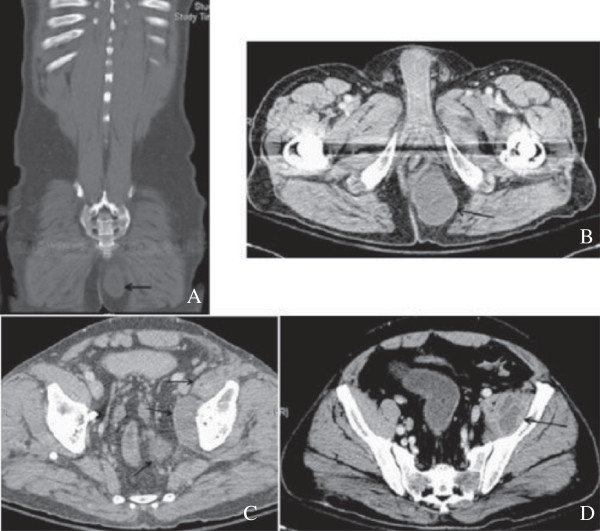
**Magnetic resonance imaging of the pelvis demonstrated large, complex, multiple lobulated abscesses with lower central attenuation and peripheral enhancement from the left ischiorectal fossa to the left iliacus muscle, iliopsoas muscle, and adductor muscle.** (**A**, **B**) One large complex abscess in the ischiorectal fossa with mass effect to the rectum. (**C**, **D**) Abscess extending to the iliopsoas muscle and iliacus muscle with multiple loculation.

**Figure 4:**
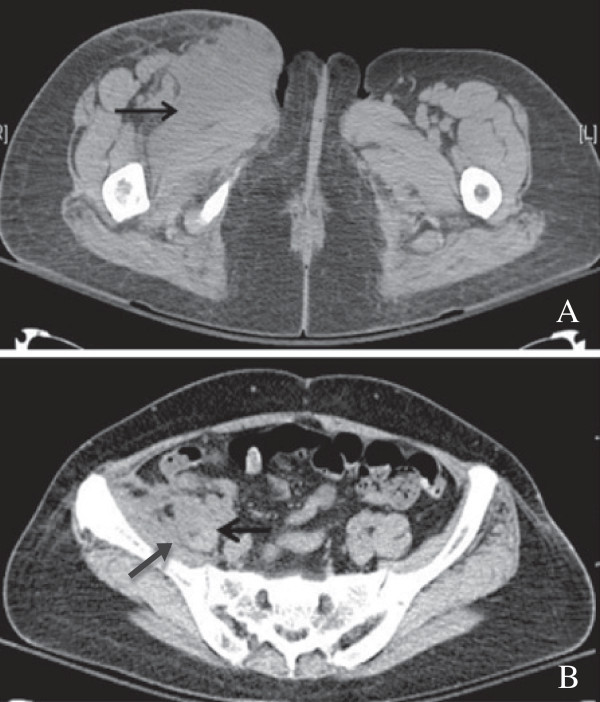
Computed tomography of the pelvis showed (A) Amorphous fluid collection with air bubbles involved proximal to the right iliopsoas muscle and distal to the adductor muscle of the right groin, (B) The right iliopsoas muscle appeared to be enlarged with an irregular margin and had some stranding adjacent to the colon.

The most significant finding of the current study was that patients with inguinal abscess of extrapelvic versus intrapelvic origin had different pathogenic findings and clinical presentations. The differentiation between these two origins could be accurately achieved by computed tomography and MRI in a timely fashion that helped guide empiric antibiotic treatment and surgical planning. Extrapelvic origin usually presented a tubular, thickened wall structure with extention to the involved musculature, such as adductor or rectus femoris. (Figures 1, 2) Intrapelvic origin presented a thicken wall with retroperitoneal or intrapertoneal extension to the involved structures (Figures 3, 4). While intrapelvic origin was identified, Group 2 patients presented with a shorter time between the admission and the first surgical intervention. This phenomenon reflected the importance of the timely imaging study as well as the more fulminant infections respresented by the higher APACHE II scores within 24 hours of admission. On the other hand, the longer time of first surgical intervention in patients with extrapelvic origin1 might result from the insidious onset of symptoms over the thigh despite imaging has shown the pathology. Although mortality rate did not increase, hospital stay became similar to those for patients with an intrapelvic origin of infection who presented higher APACHE II scores.

Patients with pyogenic inguinal abscess of extrapelvic origin presented with different predisposing comorbidities as compared with patients with abscess of intrapelvic origin. Patients with an intrapelvic origin of infection were more likely to have chronic renal failure and history of malignancy than patients with an extrapelvic origin of infection. This finding was consistent with previous reports that immunodeficiency, chronic renal insufficiency, and malignancy predisposed patients to intrapelvic infection [12,21]. Patients with an intrapelvic origin of infection had higher incidences of anemia and hyponatremiam, which might result from intra-abdominal or pelvic abscesses [22-24]. Decreased hemoglobin and serum sodium were found to be factors in poor prognosis in cases of pelvic infection [25,26]. More aggressive resuscitation, intensive care, and debridement are therefore recommended when an inguinal abscess of intrapelvic origin is suspected based on coexisting disease, clinical manifestations, and laboratory findings.

Treatment includes antibiotic therapy and surgical drainage of the abscess. Broad-spectrum antibiotic therapies should be chosen based on clinical presentation and cross sectional images in the ED. Penicillin or ampicillin plus an aminoglycoside is the typical antibiotic regimen [27]. Anaerobic coverage (metronidazole or clindamycin) is added when infection of intrapelvic origin is suspected. Computed tomography and MRI are helpful in diagnosing cases of possible abscess and determining the extent of infection [12,28].

### Limitation

The present study was limited by the small number of patients and the retrospective design. However, the occurrence of inguinal abscess was not common, with only 28 patients over an 8-year period.

## Conclusion

Computed tomography or MRI is helpful in determining the extent of the abscess and guiding the treatment plan. Higher APACHE II scores within 24 hours of hospital admission, higher incidences of anemia and hyponatremia, and a higher prevalence of chronic renal failure and history of malignancy were observed in patients with abscess of intrapelvic, as compared with extrapelvic, origin. Treatment protocols including aggressive resuscitation, rapid administration of antibiotics, and immediate surgical intervention are recommended for all patients presenting with acute pyogenic inguinal abscess.

## Competing interest

The authors declare that they have no competing interests.

## Authors’ contributions

WHH conceived the study, participated in its design and drafted the manuscript. CYL collected data and performed the statistical analysis. LJL participated in the study design and helped to draft the manuscript. TYH participated in the design of the study. KTP participated the study design and intellectual input. All authors read and approved the final manuscript.

## Pre-publication history

The pre-publication history for this paper can be accessed here:

http://www.biomedcentral.com/1471-2334/13/155/prepub

## References

[B1] Allon Amitai MaRS DO Necrotizing fasciitis as the clinical presentation of a retroperitoneal abscess J Emerg Med 2008 34 37 40 10.1016/j.jemermed.2007.03.048 17976803

[B2] Simons GW Sty JR Starshak RJ Retroperitoneal and retrofascial abscesses. A review J Bone Joint Surg Am 1983 65 1041 1058 6355110

[B3] Chankowsky JDP Gordon PH Sigmoid diverticulitis presenting as a lower extremity abscess: report of a case Dis Colon Rectum 2001 44 1711 1713 10.1007/BF02234396 11711748

[B4] Hsu SC Huang JJ Wang MC Gas-forming retroperitoneal abscess associated with crepitant myositis of right buttock and thigh J Infect 2000 40 295 297 10.1053/jinf.1999.0631 10908031

[B5] Ushiyama T Nakajima R Maeda T Perforated appendicitis causing thigh emphysema: a case report J Orthop Surg (Hong Kong) 2005 13 93 95 15872410 10.1177/230949900501300118

[B6] Donovan PJ Zerhouni EA Siegelman SS cT of the psoas compartment of the retroperitoneum Semin Roentgenol 1981 16 241 250 10.1016/0037-198X(81)90024-9 7313704

[B7] Nadeem RD Hadden WA Inguinal abscess: an unusual presentation of infection around total hip replacement J Arthroplasty 1999 14 630 632 10.1016/S0883-5403(99)90088-8 10475565

[B8] Benfatto G Catania G Tenaglia L Abscess and cecum carcinoma in inguinal hernia: case report G Chir 2006 27 262 264 17062196

[B9] Ikeda S Takeda H Yoshimitsu M Abscess in the inguinal hernial sac after peritonitis surgery: a case report World J Gastroenterol 2009 15 1007 1009 10.3748/wjg.15.1007 19248203 PMC2653403

[B10] Jaiswal P Challacombe B Dasgupta P Groin abscess: a vesico-cutaneous fistula to the groin. A rare complication of open prostatectomy Int J Clin Pract Suppl 2005 147 113 114 15875645 10.1111/j.1368-504x.2005.00303.x

[B11] Rivera-Herrera JL Otheguy JN Nieves-Ortega J Painful inguinal mass: uncommon presentation of a retroperitoneal abscess Bol Asoc Med P R 1991 83 402 403 1807275

[B12] Huang JJ Ruaan MK Lan RR Acute pyogenic iliopsoas abscess in Taiwan: clinical features, diagnosis, treatments and outcome J Infect 2000 40 248 255 10.1053/jinf.2000.0643 10908019

[B13] Bhosale PR Patnana M Viswanathan C The inguinal canal: anatomy and imaging features of common and uncommon masses Radiographics 2008 28 819 835 quiz 913 10.1148/rg.283075110 18480486

[B14] Isaacs LE Felsenstein CH Acute appendicitis in a femoral hernia: an unusual presentation of a groin mass J Emerg Med 2002 23 15 18 10.1016/S0736-4679(02)00455-9 12217466

[B15] Kolias AG Nikolaou S Bilal KO Chronic groin sinus: an unusual complication of sterilisation clips Ann R Coll Surg Engl 2010 92 W13 W14 20056050 10.1308/147870810X476638 PMC5696818

[B16] Marsh F Rogerson L Groin abscess secondary to trans obturator tape erosion: Case report and literature review Neurourol Urodyn 2007 26 543 546 10.1002/nau.20239 17304527

[B17] Ray K Tonsi AE Woods WG An isolated lump in right groin: an unusual initial presentation of Crohn’s disease Acta Chir Belg 2009 109 98 100 19341206 10.1080/00015458.2009.11680381

[B18] Ryan SP Harte PJ Suppurative inflammation of vas deferens: an unusual groin mass Urology 1988 31 245 246 10.1016/0090-4295(88)90151-3 3347975

[B19] Saha S Wright G Arulampalam T An unusual groin mass. Seminal vesicle abscess: a case report Cases J 2009 2 6531 10.1186/1757-1626-2-6531 19829819 PMC2740224

[B20] Tsai HL Hsieh JS Yu FJ Perforated colonic cancer presenting as intra-abdominal abscess Int J Colorectal Dis 2007 22 15 19 16625373 10.1007/s00384-006-0097-6

[B21] Hosseini SJ Rahmani M Razzaghi M Fournier gangrene: a series of 12 patients Urol J 2006 3 165 170 17559034

[B22] Navarro JF Mora-Fernandez C Hoffmann G Erythropoietin response is blunted in critically ill patients Intensive Care Med 1997 23 920 921 9310814

[B23] Brook I Microbiology and management of abdominal infections Dig Dis Sci 2008 53 2585 2591 10.1007/s10620-007-0194-6 18288616

[B24] Czymek R Hildebrand P Kleemann M New insights into the epidemiology and etiology of Fournier’s gangrene: a review of 33 patients Infection 2009 37 306 312 10.1007/s15010-008-8169-x 19629386

[B25] Laor E Palmer LS Tolia BM Outcome prediction in patients with Fournier’s gangrene J Urol 1995 154 89 92 10.1016/S0022-5347(01)67236-7 7776464

[B26] Yeniyol CO Suelozgen T Arslan M Fournier’s gangrene: experience with 25 patients and use of Fournier’s gangrene severity index score Urology 2004 64 218 222 10.1016/j.urology.2004.03.049 15302463

[B27] Elliott D Kufera JA Myers RA The microbiology of necrotizing soft tissue infections Am J Surg 2000 179 361 366 10.1016/S0002-9610(00)00360-3 10930480

[B28] Cantasdemir M Kara B Cebi D Computed tomography-guided percutaneous catheter drainage of primary and secondary iliopsoas abscesses Clin Radiol 2003 58 811 815 10.1016/S0009-9260(03)00274-5 14521893

